# Targeting plasmodium α-tubulin-1 to block malaria transmission to mosquitoes

**DOI:** 10.3389/fcimb.2023.1132647

**Published:** 2023-03-16

**Authors:** Genwei Zhang, Guodong Niu, Diana Hooker–Romera, Sadeq Shabani, Julian Ramelow, Xiaohong Wang, Noah S. Butler, Anthony A. James, Jun Li

**Affiliations:** ^1^ Department of Chemistry and Biochemistry, University of Oklahoma, Norman, OK, United States; ^2^ Department of Biological Sciences, Biomolecule Sciences Institute, Florida International University, Miami, FL, United States; ^3^ Biomedical Sciences Graduate Program, Herbert Wertheim College of Medicine, Florida International University, Miami, FL, United States; ^4^ Departments of Microbiology and Immunology, University of Iowa, Iowa City, IA, United States; ^5^ Department of Microbiology & Molecular Genetics and Molecular Biology & Biochemistry, University of California, Irvine, Irvine, CA, United States

**Keywords:** mosquito, pathogen-host interaction, ookinete, invasive apparatus, malaria transmission-blocking vaccine, FREP1

## Abstract

*Plasmodium* ookinetes use an invasive apparatus to invade mosquito midguts, and tubulins are the major structural proteins of this apical complex. We examined the role of tubulins in malaria transmission to mosquitoes. Our results demonstrate that the rabbit polyclonal antibodies (pAb) against human α-tubulin significantly reduced the number of *P. falciparum* oocysts in *Anopheles gambiae* midguts, while rabbit pAb against human β-tubulin did not. Further studies showed that pAb, specifically against *P. falciparum* α-tubulin-1, also significantly limited *P. falciparum* transmission to mosquitoes. We also generated mouse monoclonal antibodies (mAb) using recombinant *P. falciparum* α-tubulin-1. Out of 16 mAb, two mAb, A3 and A16, blocked *P. falciparum* transmission with EC_50_ of 12 μg/ml and 2.8 μg/ml. The epitopes of A3 and A16 were determined to be a conformational and linear sequence of EAREDLAALEKDYEE, respectively. To understand the mechanism of the antibody-blocking activity, we studied the accessibility of live ookinete α-tubulin-1 to antibodies and its interaction with mosquito midgut proteins. Immunofluorescent assays showed that pAb could bind to the apical complex of live ookinetes. Moreover, both ELISA and pull-down assays demonstrated that insect cell-expressed mosquito midgut protein, fibrinogen-related protein 1 (FREP1), interacts with *P. falciparum* α-tubulin-1. Since ookinete invasion is directional, we conclude that the interaction between *Anopheles* FREP1 protein and *Plasmodium α*-tubulin-1 anchors and orients the ookinete invasive apparatus towards the midgut PM and promotes the efficient parasite infection in the mosquito.

## Introduction

Despite significant advances in malaria control at the start of this century, cases and deaths caused by *Plasmodium* parasites remain very high and are increasing (Jagannathan and Kakuru, 2022). The rapid spread of insecticide-resistant mosquitoes and drug-resistant parasites (Yuan et al., 2011) and the limited roll-out of the modestly efficacious malaria vaccine RTS,S (Olotu et al., 2016) demand new targets and new approaches to malaria control. Infection, differentiation, and development of *Plasmodium* parasites in anopheline mosquitoes are essential for maintaining pathogen transmission. Thus, understanding the mechanisms of pathogen infection in mosquitoes could inform novel disease control approaches.

Human malaria pathogens include five *Plasmodium* species, with *P. falciparum* and *P. vivax* responsible for 99% of malaria cases (Kassa et al., 2006; Chery et al., 2016; Qureshi et al., 2019). Mosquitoes in the genus *Anopheles* are the only vectors of human pathogens. When a mosquito feeds on a *Plasmodium*-infected person, gametocytes in the blood enter the mosquito midgut, where haploid microgametes (male-equivalent germ cell) and macrogametes (female-equivalent germ cell) fertilize to form diploid zygotes. The zygotes quickly develop into motile ookinetes. Ookinetes must overcome the physical barriers of the mosquito midgut, the peritrophic matrix (PM), and the apical surface of the midgut epithelium to establish an infection. Once the ookinetes reside between the midgut epithelium and basal lamina, they differentiate into oocysts. A series of mitotic divisions follow meiosis to give rise to many sporozoites. These developmental forms make their way to the mosquito salivary glands, where they can infect a new host during subsequent blood-feeding.

The *Plasmodium* ookinete has an asymmetrical oval shape with distinct apical and basal ends. An invasive apparatus at the apical end secretes digestive enzymes that disrupt the physical barriers inside midguts (Patra and Vinetz, 2012). Thus, orienting and anchoring the ookinete apical end towards mosquito midgut epithelium is necessary for ookinete penetration of the PM and infection of the epithelial cells. The apical polar complex comprises tubulins and other proteins and undergoes a dynamic structural change, e.g., protrusion and conoid around apical rings, during *Plasmodium* invasion of host cells (Bertiaux et al., 2021). *Plasmodium* tubulin proteins include α-tubulin-1, α-tubulin-2, and β-tubulin (Rawlings et al., 1992). Only α-tubulin-1 and β-tubulin are expressed in diploid ookinetes. The α-tubulin-2 is expressed abundantly in the male gametocytes but is present at low levels in female gametocytes or ookinetes (Kooij et al., 2005; Yeoh et al., 2017).

Several molecular interactions critical for productive infection occur between the mosquito and parasites in the midgut. For example, *Plasmodium vivax* Pvs25 binds to calreticulin, a mosquito midgut apical surface protein (Rodriguez Mdel et al., 2007). An ookinete surface enolase interacts with a midgut enolase-binding protein to mediate the invasion of *P. berghei* but not *P. falciparum* (Vega-Rodriguez et al., 2014). Notably, none of these interactions appear conserved across multiple species of *Plasmodium* and *Anopheles*. Thus, discovering a conserved pathway for parasite infection and subsequent development may provide a broad-spectrum target for interrupting pathogen transmission through mosquitoes. Antimalarial vaccine efforts have mainly focused on merozoite and sporozoite surface proteins (Neal et al., 2010; Yoshida et al., 2010; Alaro et al., 2013; Burns et al., 2016; Sheikh et al., 2016; Laurens, 2020). The RTS,S vaccine, based on *P. falciparum* circumsporozoite protein and introduced in 2019 (Laurens, 2020), is the only malaria vaccine available. However, it provides modest efficacy, ~ 30% (Syed, 2022). Therefore, the malaria control community still needs targets for vaccine and transmission-blocking vaccine (TBV) development (Sinden and Billingsley, 2001).

The *FREP1* gene was discovered for its significant correlation in modulating the infection intensity of clinically circulating *P. falciparum* in wild *An. gambiae* in Kenya (Li et al., 2013). Further investigation revealed that the gene product, FREP1, was a component of the mosquito midgut PM that bound *P. falciparum* ookinetes to facilitate their invasion (Zhang et al., 2015). Experiments with transgenic *An. gambiae* (Dong et al., 2018) and transmission-blocking small molecule discovery approaches (Niu et al., 2015) established a critical role for FREP1 in *Plasmodium* transmission. Notably, pAb against FREP1 could inhibit multiple species of *Plasmodium* (*P. falciparum*, *P. vivax*, and *P. berghei*) from invading multiple species of *Anopheles* (e.g., *An. gambiae* and *An. dirus*) (Niu et al., 2017), supporting the conclusion that FREP1-mediated *Plasmodium* infection pathways are conserved highly across *Plasmodium* and *Anopheles* (Niu et al., 2017). Despite the importance of the FREP1-mediated *Plasmodium* invasion pathway, the parasite-expressed FREP1-binding partners (FBPs) had not been identified. Here, we focus on the functional characterization of *Plasmodium* α-tubulin-1 related to *Plasmodium* infection of mosquitoes and its interaction with FREP1. We demonstrated that they could be targeted to disrupt the *Plasmodium* infection of mosquitoes.

## Results

### Rabbit pAb against human-tubulin inhibited *P. falciparum* transmission to mosquitoes

**Figure 1:**
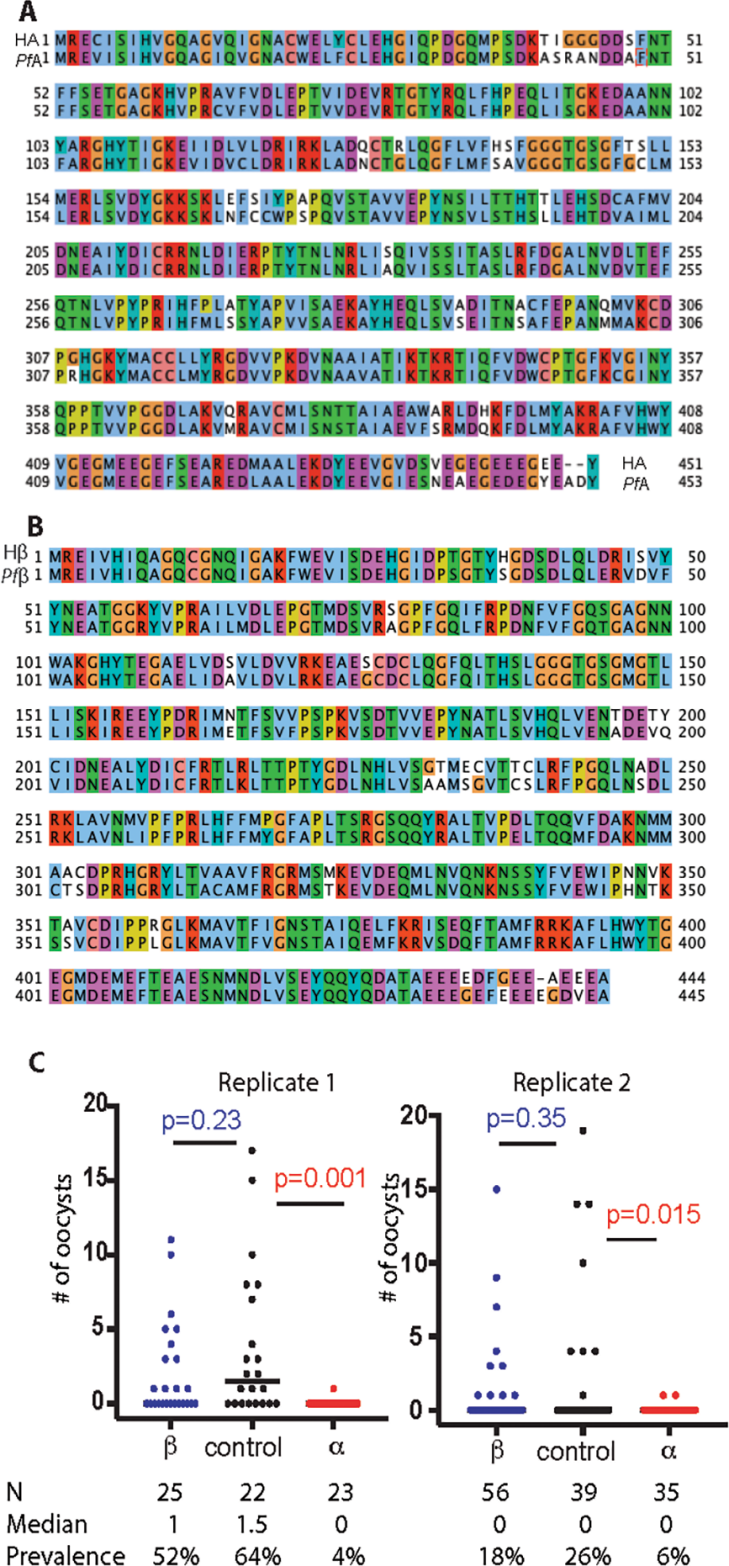
Tubulin proteins are highly conserved between human and *Plasmodium* and the effects of polyclonal anti-tubulin antibodies on oocyst development. **(A)** Sequence alignment of human α-tubulin (HA) and *P. falciparum* α-tubulin-1 (*Pf*A). Clustal X Colour Scheme was used to show amino acids. **(B)** Sequence alignment of human β-tubulin (Hβ) and *P. falciparum* β-tubulin-1 (*Pf*β). Clustal X Colour Scheme was used to show amino acids. **(C)** Standard membrane feeding assays were used to measure Intensity and prevalence of oocysts in the presence of purified rabbit anti-human α-tubulin polyclonal Ab (labeled with α, 0.01 mg/ml ) and purified polyclonal Ab against human β-tubulin (labeled with β, 0.01 mg/ml). A non-related purified rabbit polyclonal Ab (anti-V5, labeled with control, 0.01 mg/ml) was used as the negative control. Mann-Whitney test was used to calculate P-value. N, number of mosquitoes.

The *P. falciparum* α-tubulin-1 and β-tubulin are two components of the miroctubles and polar rings in the invasive apparatus. *P. falciparum* α-tubulin-1 and human α-tubulin sequences share >84% amino acid identity and 93% (422/453) sequence similarity (**Figure 1**). *P. falciparum* β-tubulin-1 and human β-tubulin sequences share >85% amino acid identity and 94% (417/445) sequence similarity (**Figure 1**). Therefore, the capacity of purified polyclonal anti-human *α*-tubulin and β-tubulin Ab to block *P. falciparum* oocyst formation in mosquitoes was examined using the standard membrane feeding assays (SMFA) (Zhang et al., 2015). These assays determine efficacy by counting the number of oocysts (intensity of infection) and prevalence of infection formed in midguts with and without experimental interventions. An irrelevant rabbit pAb (anti-V5) was used as a negative control. The mortalities of mosquitoes fed with or without pAb were below 10%, e.g., 0 to 2 dead mosquitoes per treatment, displaying no difference. The results show that pAb (0.01 mg/ml) against human α-tubulin significantly reduced the number of *P. falciparum* oocysts in *An. gambiae* midguts (*p*=0.015), compared to controls (**Figure 1**). The infection prevalence decreased from 26% and 64% to 6% and 4% in two replicates. Oocysts were detectable in only one or two mosquitoes from the anti-α-tubulin experimental feeding groups. On the contrary, there is no significant difference in oocyst counts between pAb against human β-tubulin and the control mosquito midguts (p=0.23 and 0.35), indicating that the pAb against human β-tubulin did not prevent ookinetes from invading midguts (**Figure 1**). Collectively, these data support the conclusion that α-tubulin-1 in sexual stage parasites plays key roles during malaria transmission to mosquitoes and is accessible to extracellular antibodies.

### Antibodies against human-tubulin do not affect the conversion of *P. falciparum* gametocytes to ookinetes

A lower gametocyte-to-ookinete conversion might cause the Ab-mediated reduction of oocysts. Therefore, we tested this possibility. The purified rabbit polyclonal Ab against human α-tubulin was added into the *P. falciparum* cultures containing 3-10% gametocytes. An equal amount of non-related purified rabbit Ab (anti-V5) was used as a negative control. After incubation, the gametocytes and ookinetes were stained with the Giemsa and examined microscopically. A statistically equivalent number of gametocytes and ookinetes were detected in both the experimental and control groups. The ookinete conversion rates (CR, calculated as the percentage of ookinetes among the total gametocytes) in the presence of anti-α-tubulin Ab and anti-V5 Ab (negative control) are shown in **Table 1**. Three replicates showed no significance between the experimental and control groups (p=1). These data support the conclusion that Ab against α-tubulin-1 does not affect the conversion of *P. falciparum* gametocytes to ookinetes.

**Table 1:** The effects of antibodies on ookinete conversion.

Dilution	Antibody	Replicates	Gametocytes	Ookinetes	Conversion Rate (%)	P value
50-fold	Anti-human α-tubulin	1	50	8	16.0	1
		2	62	6	9.7	
		3	56	8	14.3	
	Anti-V5	1	52	8	15.4	
		2	60	6	10	
		3	52	7	13.5	
30-fold	Anti-human α-tubulin	1	22	3	13.6	1
		2	16	5	31.2	
		3	19	4	21.0	
	Anti-V5	1	23	4	17.4	
		2	19	4	21.1	
		3	16	3	18.9	

The two dilutions were conducted with different *P. falciparum* cultures. P values were calculated by Wilcoxon test in R.

### Customized rabbit pAb against *P. falciparum* α-tubulin-1 significantly reduced Plasmodium infection in mosquitoes

Next, we used commercially generated specific pAb against *P. falciparum* 3D7 α-tubulin-1 to examine their effects on *P. falciparum* transmission. The *P. falciparum* 3D7 *Hsp70* gene was chosen as a negative control because it is also an abundant cytoplasmic protein. The pAb against *P. falciparum* α-tubulin-1 was generated in rabbits.

**Figure 2:**
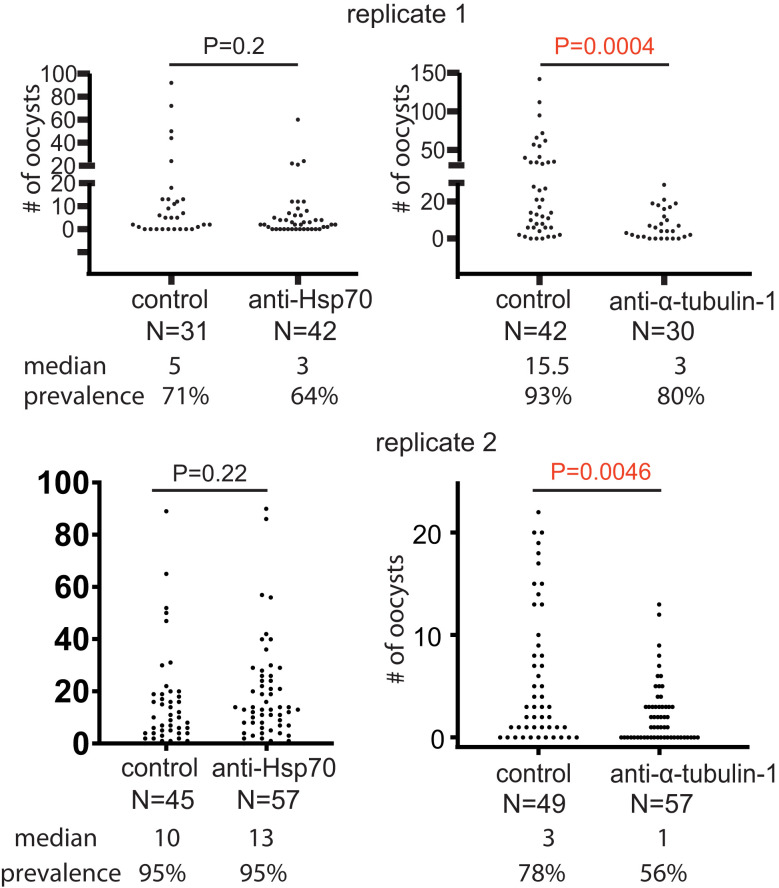
Anti-α-tubulin-1 antisera block *P. falciparum* transmission to *An. gambiae*. Infection intensity and prevalence of oocyst development in the presence of anti-α-tubulin-1 (titer: 1x10^5^), anti-HSP70 (control, titer: 1x10^5^), and the control (pre-immune serum) by standard membrane feeding assays. P values were calculated using the Mann-Whitney test. N, number of mosquitoes.

Since antibody titers in sera are related to antibody’s interactivity with antigens, the anti-sera were normalized with a pre-immune serum to the titer of 2x10^5^ (about 5-10 folds dilution). The sera were mixed with the same volume of cultured *P. falciparum* and used to infect *An. gambiae* with SMFA, as described previously. Pre-immune serum served as a negative control. The results showed that antiserum against α-tubulin-1 significantly (p=0.0046 and 0.0004 in two replicates) reduced by 3-5-fold the median intensity of infection of *P. falciparum* oocysts in mosquitoes in the two independent replicates when compared to the pre-immune serum (**Figure 2**). The antisera against the Hsp70 did not significantly impact *P. falciparum* infection intensity (*p*=0.20 and 0.22 in two replicates).

**Figure 3:**
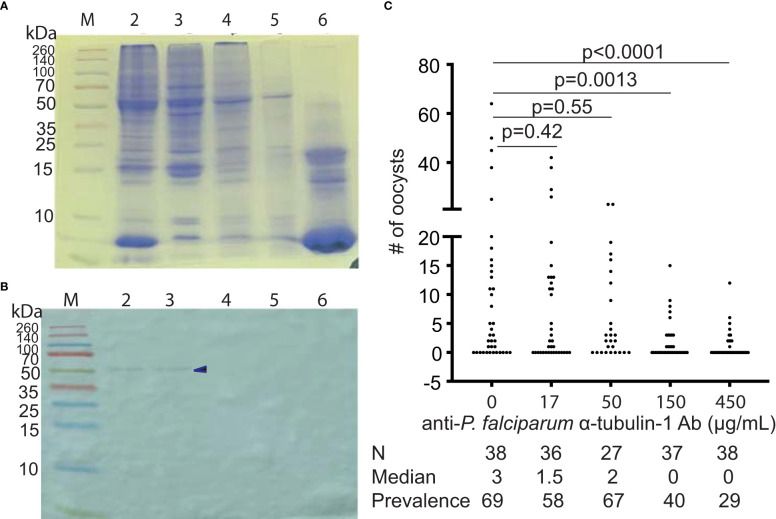
Purified rabbit pAb recognizes *P. falciparum* α-tubulin-1 and inhibits the *P. falciparum* transmission to *An. gambiae*. **(A)** Proteins in cell lysates were separated by SDS-PAGE and **(B)** analyzed by immunoblotting showing the purified rabbit pAb recognize *P. falciparum* α-tubulin-1. Lanes, M: protein ladder; 2: *P. falciparum* lysate; 3: α-tubulin-1 expressed Hi5 lysate; 4: Hi5 lysate; 5: HEK 293 cell lysate. 6: Whole blood lysate. **(C)** The number of oocysts (infection intensity) in individual mosquitoes and prevalence in the presence of purified rabbit anti-*P. falciparum* α-tubulin-1 Ab at different concentrations (0,17, 50, 150, and 450 μg/ml) by SMFA. N, number of mosquitoes. P values were calculated by Mann-Whitney test.

We purified anti-*P. falciparum* α-tubulin-1 pAb to remove the serum factors. First, we analyzed the specificity of the purified antibodies. The SDS-PAGE was used to separate proteins in the lysates of cultured *P. falciparum* with ookinetes, *P. falciparum* α-tubulin-1-expressed Hi5, Hi5, human liver cells (HEK 293), and whole blood (**Figure 3**). Immunoblotting analyses with the purified rabbit pAb specifically recognized α-tubulin-1 in *P. falciparum* (**Figure 3**). Moreover, the transmission-blocking activity in SMFA of this Ab at different concentrations from 0 to 450 μg/mL against *P. falciparum* showed significantly reduced oocysts in numbers in a concentration-dependence (**Figure 3**). As the concentration of Ab increased, the median number of oocysts and infection prevalence decreased from 3 and 69 to 0 and 29, respectively. The half-maximal inhibitory concentration (IC_50_) is calculated to be 36 μg/mL.

### Examining a panel of mAb against *P. falciparum* α-tubulin-1

**Figure 4:**
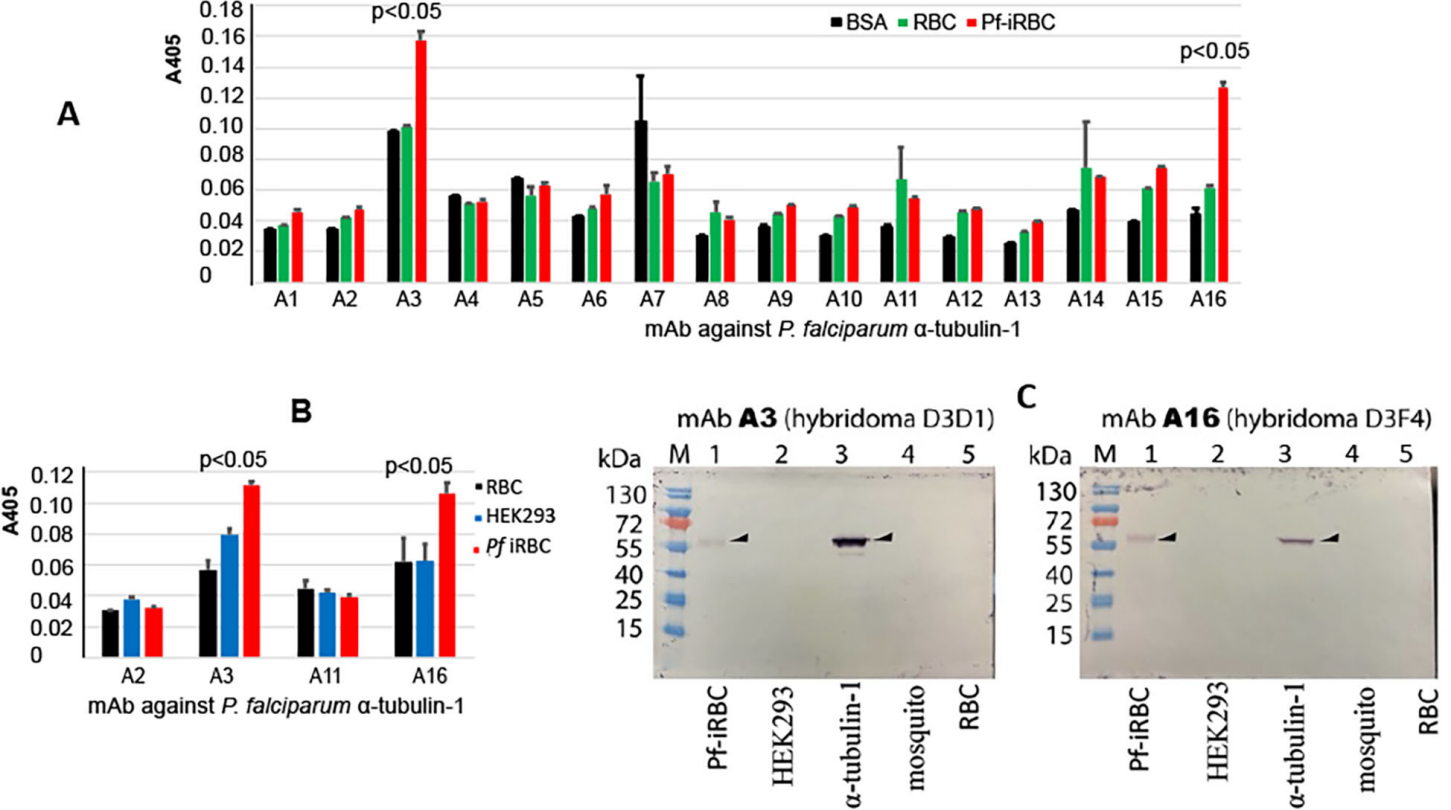
Different mAb show different reactions. **(A)** ELISA show the reactions of 16 mAb to samples. The data exhibit means and standard deviations from three replicates. A3 and A16 bind to Pf-iRBC lysate significantly. **(B)** Binding assay of four mAb confirm the interaction A3 and A16 with *Pf*-iRBC. A3 has some degree of cross-reaction with human kidney cell (HEK293) lysate. The data display means and standard deviations from three replicates. **(C)**: Specificity analyses of two anti*-*α-tubulin-1 mAb by Western blotting show that A3 and A16 recognize *P. falciparum* α-tubulin-1 specifically.

Since the sequence between human α-tubulin and *P. falciparum* α-tubulin-1 share ~ about 84% identity, mAb was generated to evaluate the specific reaction and cross-reaction. The *E. coli*-expressed *P. falciparum* α-tubulin-1 was used to immunize mice. Sixteen hybridomas were generated that produced mAb (mAb) (Boster Bio, Pleasanton, CA). Using ELISA, the molecular interaction between mAb and bovine serum album (BSA), the lysates of uninfected human red blood cells (RBC), and *P. falciparum*-infected human red blood cells (*Pf*-iRBC) showed that two mAb (A3 and A16) bound to *Pf*-iRBC specifically (**Figure 4**). The other fourteen mAb did not bind to *Pf*-iRBC lysate.

We evaluated the cross-reaction of mAb with human red blood cell lysate and human kidney cell (HEK293) lysates. A3 and A16 were confirmed to interact with *Pf*-iBRC lysate (**Figure 4**). Also, A16 did not interact with RBC or HEK293 lysates, while A3 displayed a slight degree of cross-reaction with HEK293 lysate (p=0.1). Therefore, we used a Western blot to confirm the specificity of the mAb to tubulins in the lysate of *P. falciparum*-infected red blood cells (*Pf*-iRBC), human kidney cells (HEK293), recombinant *P. falciparum* α-tubulin-1 protein, mosquitoes, and human red blood cells (RBC). As expected, both A3 and A16 recognized α-tubulin-1 in *P. falciparum*-infected red blood cells and recombinant α-tubulin-1 protein from Hi5 cells (**Figure 4**). Notably, they did not bind to tubulins in human cells and mosquitoes.

**Figure 5:**
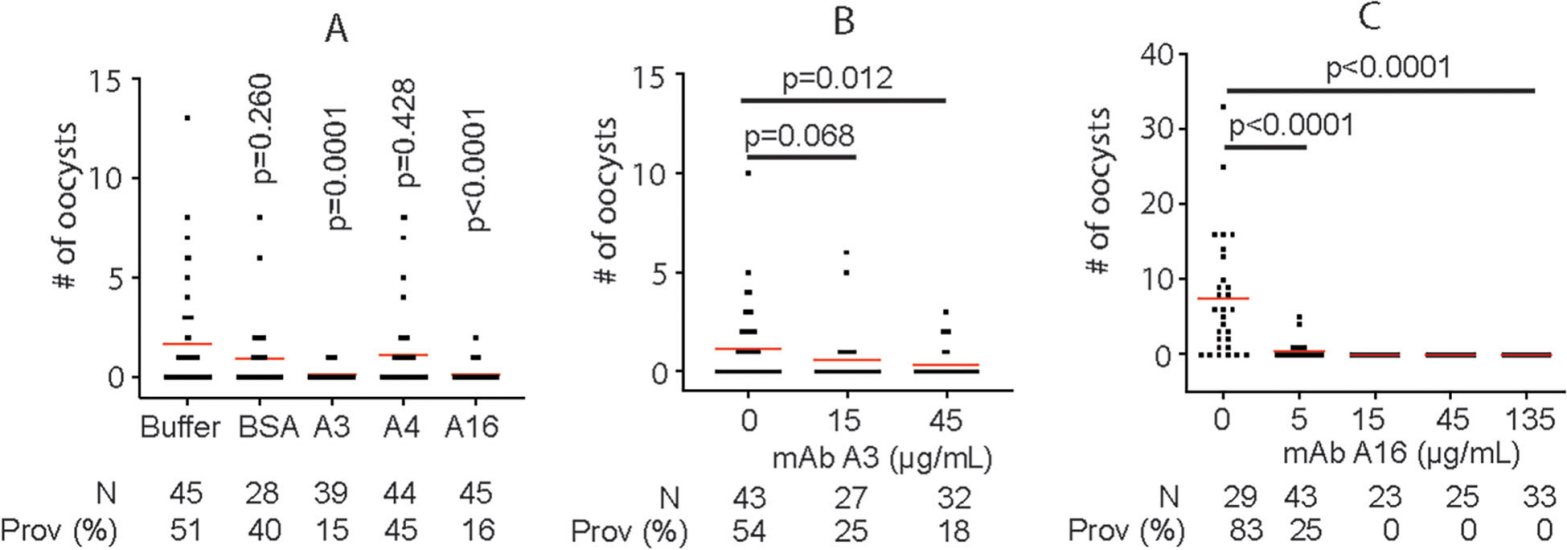
MAb A3 and A16 inhibit *P. falciparum* infection in *An. gambiae.* **(A)** SMFA show that A3 and A16 significantly inhibit *P. falciparum* transmission to mosquitoes. **(B)** The transmission-blocking activities of A3 depend on the antibody’s concentration. **(C)** A16 shows concentration-dependent transmission-blocking activity. Prov (%): prevalence. Each dot represents the number of oocysts from one individual mosquito. Red lines show the average number of oocysts per midgut. P values were calculated by Mann-Whitney tests.

Next, we examined the efficiency of mAb in blocking *P. falciparum* transmission to *An. gambiae* by SMFA. Results showed that both A3 and A16 mAb (final concentration was ~ 10 μg/mL) significantly inhibited malaria transmission (**Figure 5**). At the same time, mAb A4 did not specifically bind to *P. falciparum* lysate nor inhibit malaria transmission. Furthermore, the efficiency of mAb in blocking *P. falciparum* transmission to *An. gambiae* was examined using diluted mAb. Results confirmed that both A3 and A16 mAb significantly inhibited malaria transmission. A3 and A16 mAb inhibited *P. falciparum* transmission to *An. gambiae* with IC_50_ of 12.2 μg/ml (**Figure 5**) and 2.8 μg/ml (**Figure 5**), respectively.

### Analysis of epitopes of mAbs

**Figure 6:**
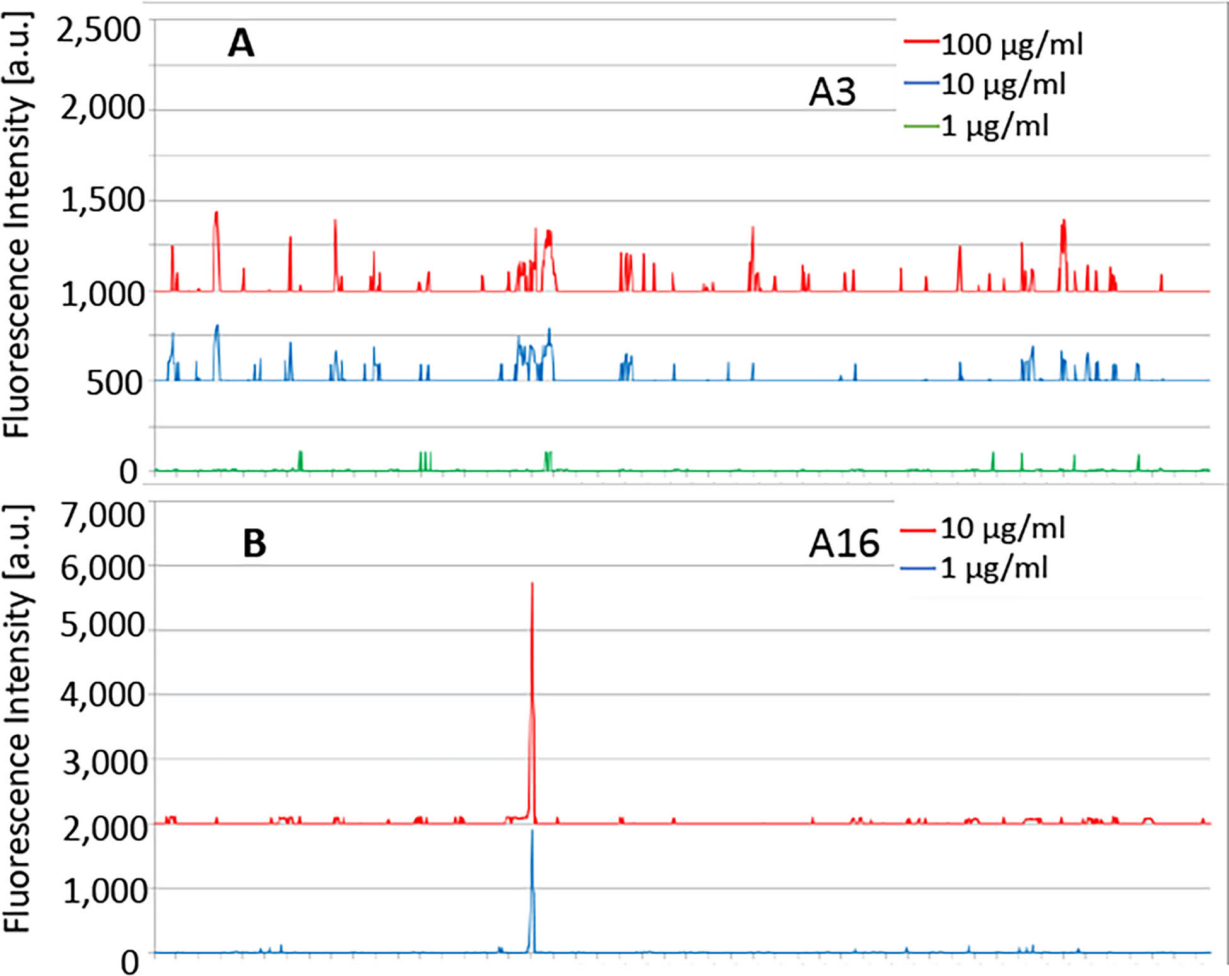
The customized peptide arrays map epitopes of A3 and A16. **(A)** No specific linear epitopes were detected for A3. **(B)** The epitope of A16 was detected. Strong monoclonal antibody response against a single epitope-like spot pattern formed by adjacent peptides with the consensus motif REDLAALEKD on α-tubulin-1.

The sequence of *P. falciparum* α-tubulin-1 (*P. falciparum* 3D7; XP_001351911.1) was elongated with neutral GSGSGSG linkers at the C- and N-terminus to avoid truncated peptides. The elongated antigen sequences were converted into 15 amino acid peptides with a peptide-peptide overlap of 14 amino acids. The resulting custom peptide microarrays contained 1,191 different peptides printed in duplicate (2,382 peptide spots) and were framed by additional HA (YPYDVPDYAG, 106 spots) control peptides. Incubation of a custom peptide microarray with mouse IgG antibody A3 (D3D1) at concentrations of 1 µg/ml, 10 µg/ml, and 100 µg/ml in incubation buffer was followed by staining with the secondary and control antibodies (red = sample staining/green = control staining). We did not observe any strong interaction signals, suggesting that mAb A3 recognized a conformational epitope or an epitope formed by a peptide longer than 14 amino acids (**Figure 6**).

We also mapped the epitope of mAb A16 (D3F4). Peptide microarray with mouse A16 mAb at concentrations of 1 µg/ml and 10 µg/ml in incubation buffer was followed by staining with the secondary and control antibodies. We observed a strong mAb response against a single epitope-like spot pattern formed by adjacent peptides with the consensus motif RED**L**AALEKD on α-tubulin-1 at high signal-to-noise ratios (**Figure 6B**). This sequence is one amino acid (bold L to M) difference at the center of this epitope between *P. falciparum* α-tubulin-1 and human α-tubulin.

### Anti-tubulin-1 pAb binds to the apical complex of a live *P. falciparum* ookinete

**Figure 7:**
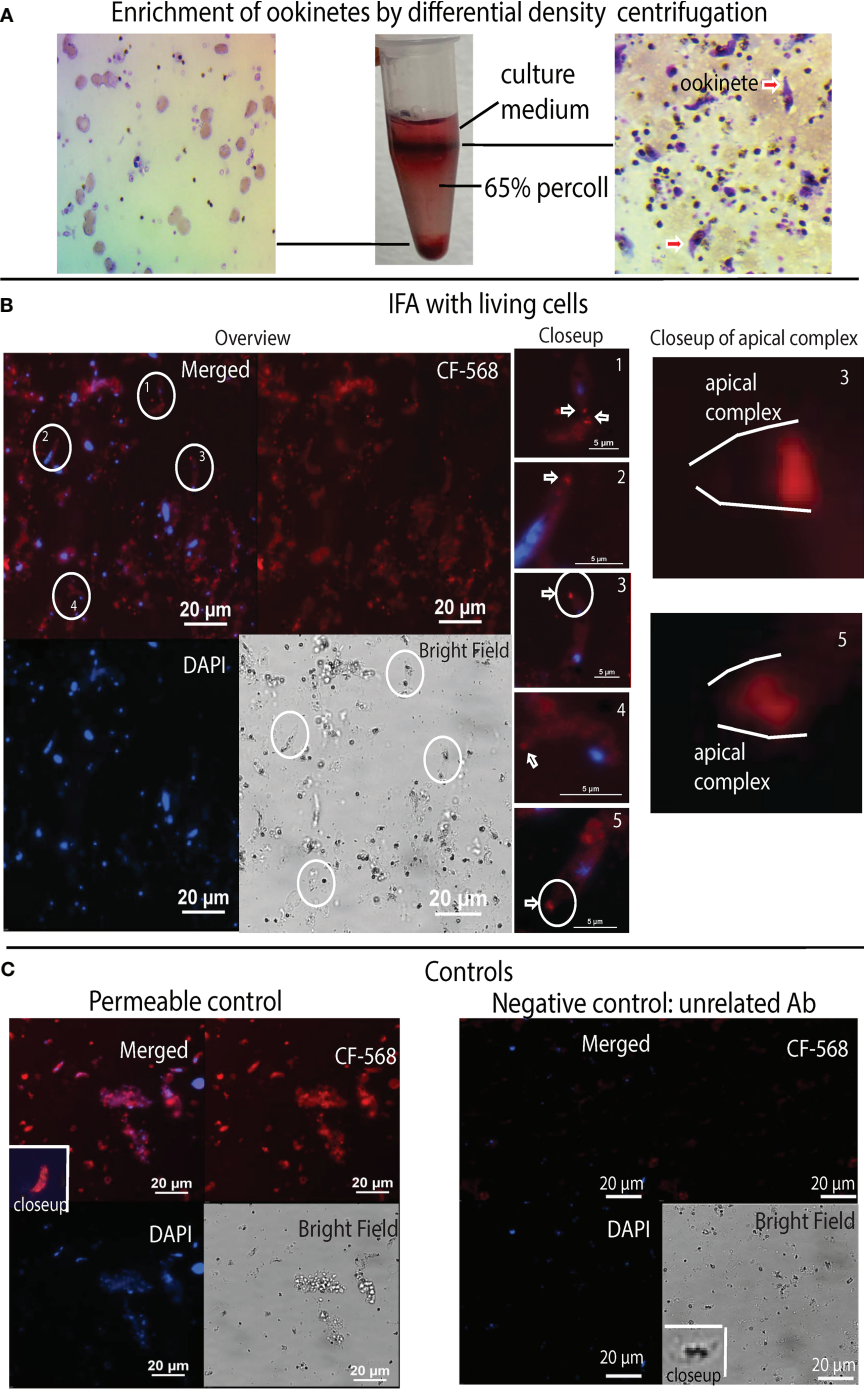
Anti-α-tubulin Ab binds live *P. falciparum* ookinetes at their apical end. **(A)** Enriching ookinetes through differential density centrifugation using 65% Percoll. Arrows point ookinetes. **(B)** IFA assays localized α-tubulin-1 on living ookinetes. The co-localization of *P. falciparum* (nuclei, blue color) and α-tubulin-1 (red). *A closeup* of individual ookinetes shows Ab bound to the apical end of living *P. falciparum* ookinetes. *A closeup of the apical complex* shows anti-α-tubulin Ab bound to the apical polar ring of living ookinetes. **(C)** Ookinetes stained with CF568 dye-conjugated anti-V5 antibodies as a negative control showed no binding. Ookinetes fixed by methanol stained with anti-α-tubulin Ab, showing that Ab could stain α-tubulin-1 tubulin inside the permeable cells.

We also determined the interaction between antibodies against *P. falciparum* α-tubulin-1 and impermeable ookinetes. We labeled customized polyclonal rabbit anti-α-tubulin-1 Ab with CF™ 568 dye and examined the antibody binding sites on live ookinetes under a fluorescence microscope. *P. falciparum* ookinetes were cultured *in vitro* (Zhang et al., 2015) and enriched by differential density centrifugation using 65% Percoll. Uninfected red blood cells and cell debris were sedimented to the bottom of the Percoll, while gametocytes and ookinetes were enriched at the interface of the Percoll and the culture medium (**Figure 7**). The enriched gametocytes and ookinetes were washed and incubated with the CF™ 568-conjugated anti-α-tubulin Ab. After removal of the unbound Ab, the cells were deposited onto coverslips and counterstained with DAPI. Fluorescence microscopy revealed that anti-α-tubulin Ab is bound to the apical end of ookinetes (**Figure 7**). Detailed examination of individual ookinetes revealed an intense fluorescent signal localized to apical ends (white arrows in **Figure 7**, *closeup*), supporting the conclusion that anti-α-tubulin Ab recognized the α-tubulin-1 of the living ookinetes at their apical ends. Notably, we also saw that anti-α-tubulin-1staining was not concentrated at the extreme distal tip of the ookinetes (**Figure 7**, *closeup of apical complex*), but instead, the signal was localized to the apical ends, suggesting that Ab recognized α-tubulin-1 at apical polar rings of living ookinetes. Importantly, fixation with methanol and permeabilization of ookinetes revealed a signal that was distributed across the cells (**Figure 7**, permeable control). Substitution of anti-α-tubulin polyclonal Ab with an irrelevant (anti-V5) polyclonal Ab revealed no signal (**Figure 7**, Negative control).

**Figure 8:**
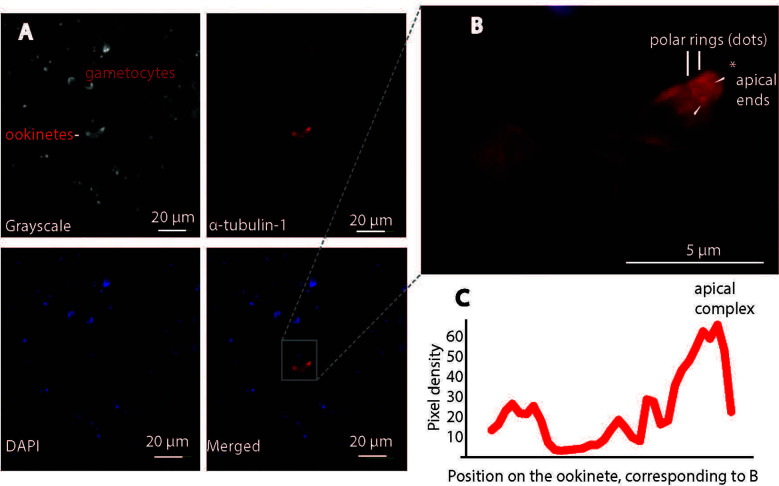
Confocal immunofluorescence assays confirm *P. falciparum* α-tubulin-1 protein on the live ookinete surface. **(A)** α-tubulin-1 was detected on the live ookinete surface and not on the gametocyte surface. **(B)** Higher magnification showing the ookinete invasion apparatus of the ookinete (protrusion) and the strongest signals (red dots) evenly distributed at the apical polar rings (pointed by white arrows). * is the apical end. **(C)** Two rings at the apical region displayed the highest pixel density of red color.

Next, we examined the cells under a confocal microscope to obtain higher-resolution images. Gametocytes were observed in the bright field, but anti-α-tubulin-1 antibodies did not bind to them (**Figure 8**), supporting the conclusion that α-tubulin-1 is not exposed at the gametocyte surface or that the host cell membrane covers gametocytes. Notably, anti-α-tubulin-1 antibodies were bound to a live ookinete (**Figure 8**). Upon higher magnification, the invasive apparatus of the ookinete could be distinguished (**Figure 8**), showing a protruding component was visible at one apical end of the ookinete. Two rings were detected at the apical complex. Indeed, the pixel intensity for the red color (α-tubulin-1) at the ookinete apical end is much stronger than in the other positions, and the apical polar rings were the strongest (**Figure 8**). These imaging studies support the conclusion that α-tubulin-1 is exposed on live ookinete surface at apical rings of the invasive apparatus.

### *P. falciparum* α-tubulin-1 interacts with *An. gambiae* midgut protein FREP1

**Figure 9:**
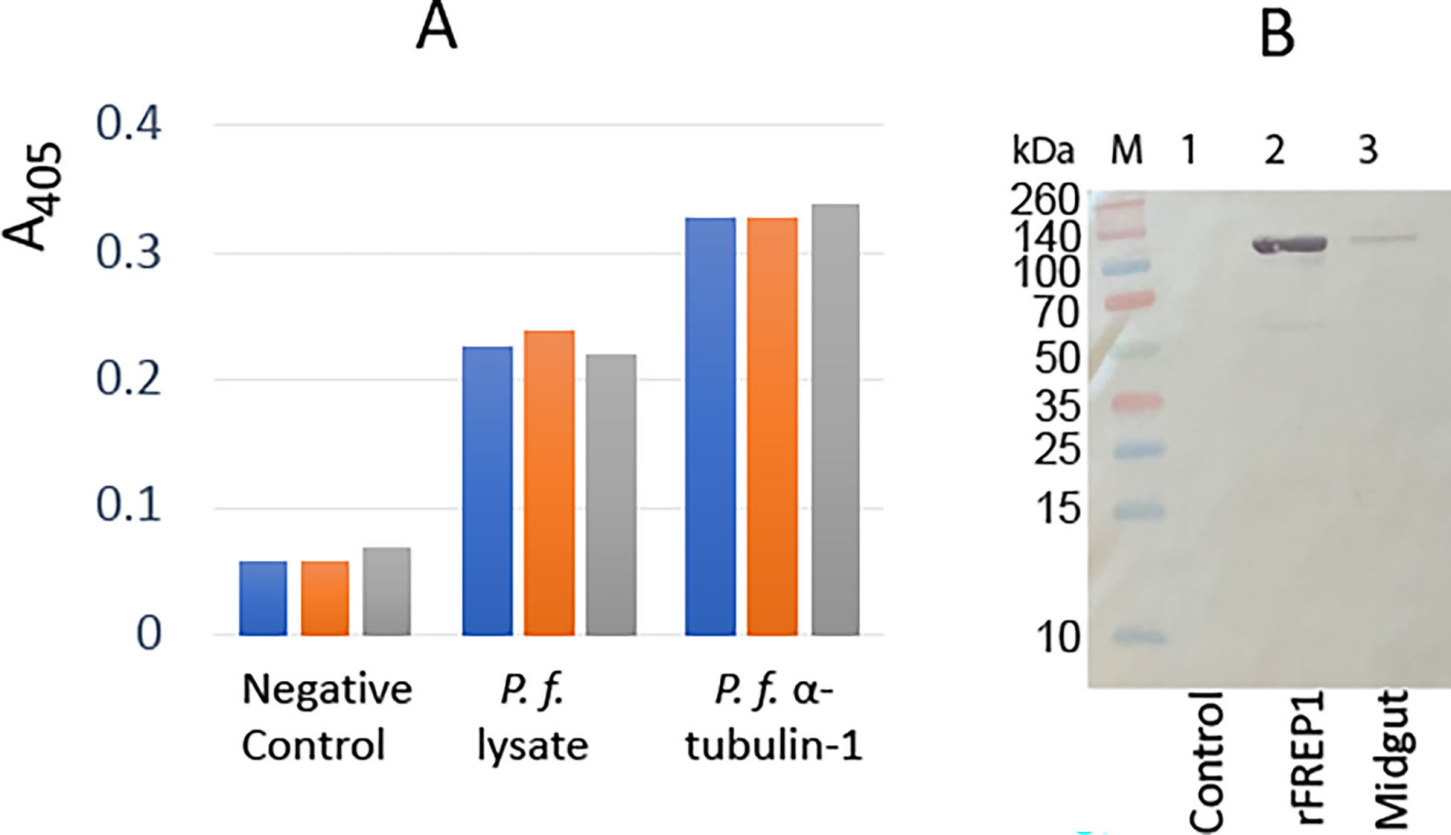
*P. falciparum* α-tubulin-1 interacts with *An. gambiae* FREP1. **(A)** ELISA showed that *P. falciparum* α-tubulin-1 bound to *An. gambiae* FREP1. A bar exhibits the actual value of A_405_ from one replicate, and there are three replicates per treatment. **(B)** *P. falciparum* α-tubulin-1 pulled down FREP1 from mosquito midgut lysate, which was detected by a western blotting assay. .

Since *P. falciparum* α-tubulin-1 in the invasive apparatus is accessible by large external molecules, we tested its interaction with *An. gambiae* midgut protein FREP1. The insect cell-expressed recombinant full-length FREP1 was used to coat the ELISA plates, followed by incubation with insect cell-expressed recombinant *P. falciparum* α-tubulin-1. The retained α-tubulin-1 was detected using a purified anti- α-tubulin-1 pAb. The insect cell-expressed chloramphenicol acetyltransferase (CAT) coated additional wells in the plates were used as the negative control. Since FREP1 can interact with *P. falciparum* lysate (Zhang et al., 2015), *P. falciparum*-infected blood cell lysates served as a positive control. Results showed that *P. falciparum* α-tubulin-1 bound FREP1 protein compared to background signals detected in the negative control wells (*p*<0.05) (**Figure 9**). A midgut lysate mixed with Hi5-expressed α-tubulin-1 with a His-tag, pulled down by Ni-NTA beads, and detected by rabbit anti-FREP1 polyclonal antibodies, showed endogenous FREP1 was detected in the α-tubulin-1-pulldown fraction (**Figure 9**), demonstrating that the strong interaction between α-tubulin-1 and FREP1 among the mixture of various proteins.

## Discussion

The *Plasmodium* ookinete has an invasive apparatus at the apical end that secretes enzymes to disrupt the physical barriers inside midguts (Patra and Vinetz, 2012). The apical polar complex comprises apical rings and a dynamic protruder during the *Plasmodium* invasion of host cells (Patra and Vinetz, 2012). *Plasmodium* tubulins form an apical tubulin ring that co-localizes with the conoid (Bertiaux et al., 2021). Our data show that α-tubulin-1 on live ookinetes can be recognized by extracellular antibodies, and targeting α-tubulin-1 by antibodies can block parasite transmission to mosquitoes.

*P. falciparum* α-tubulin-1 and β-tubulin are highly conserved with human α-tubulin and β-tubulin, respectively. Both α-tubulin-1 and β-tubulin are expressed in ookinetes (Rawlings et al., 1992). However, only α-tubulin-1 could be targeted by antibodies against α-tubulin to limit *P. falciparum* transmission to mosquitoes, while β-tubulin could not, suggesting the different functional roles between α-tubulin-1 and β-tubulin.

Moreover, we demonstrated that customized specific pAb against *P. falciparum* α-tubulin-1 inhibited *P. falciparum* oocyst development in mosquito midguts in a concentration-dependent manner. The possibility that the developmental effects of the antibody could be caused by their interference with ookinete formation was excluded by demonstrating that anti-α-tubulin Ab did not interfere with the gametocyte conversion to ookinetes.

To remove possible cross-reactions between an antibody and α-tubulin-1, we developed a panel of mAbs. We identified two specifically that bound to *P. falciparum* α-tubulin-1 and did not interact with human α-tubulin. The two mAb showed different transmission-blocking activities and recognized different epitopes. The IC_50_ of A16 mAb was <8% of pAb, and A16 recognized a linear peptide REDLAALEKD on α-tubulin-1. Notably, this sequence is different from human α-tubulin.

Accessibility of α-tubulin-1 to antibodies was confirmed using IFA on live ookinetes. Extracellular anti-α-tubulin-1 Ab bound to the apical rings of live ookinetes. As the ookinete apical end has the invasive apparatus that plays a critical role in a parasite to penetrate midgut PM and epithelium prior to oocyst development (Morrissette and Sibley, 2002; Patra and Vinetz, 2012), this result is consistent with the observed Ab-mediated reduction in oocyst prevalence and intensity of infection in the SMFA experiments. The mechanisms by which *α*-tubulin-1 becomes exposed on the ookinete surface at apical polar rings require further investigation. However, many proteins without signal peptides translocate to the ookinete surface. For example, the *P. yoelii* guanylate cyclase β (GCβ) is a cytoplasmic protein, forming a complex with CDC-50A inside cells. During the zygote-to-ookinete differentiation, this complex is translocated to the outer edge of the ookinete and exposed to the surface for ookinete gliding motility (Gao et al., 2018). More recent direct labeling evidence shows clearly that the cytoskeleton proteins myosin A and actin are exposed on the surface of sporozoites (Swearingen et al., 2016).

**Figure 10:**
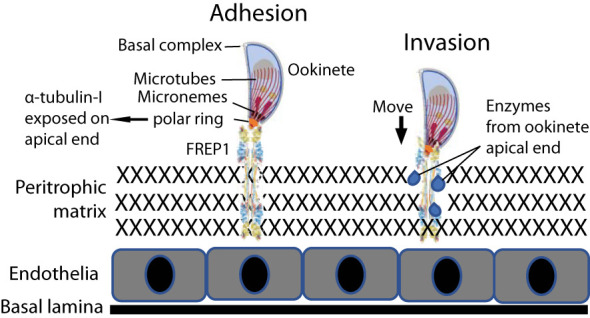
Model of the FREP1-α-tubulin-1 interaction that mediates *Plasmodium* infection of the mosquito midgut. Adhesion) The interaction between FREP1 in the peritrophic matrix (PM) and parasite α-tubulin-1 exposed on the cell surface at ookinete apical ends orientates invasive apparatus opening toward the PM. Invasion) Enzymes released from the parasitic apical opening disrupt midgut integrity. Together, these actions facilitate an ookinete to penetrate the midgut physical barrier for invasion.

The protein-protein interaction between parasites and midguts is critical in malaria transmission. Three classes of these proteins have been reported: proteases, anchors, and immunity inhibitors. Anopheline alanyl aminopeptidase N (AnAPN1) (Armistead et al., 2014) and carboxypeptidase B (Lavazec et al., 2007) are two identified proteases that promote parasite invasion in mosquitoes. *Anopheles* fibrinogen-related protein 1 (FREP1) (Zhang et al., 2015) and the Pfs47 receptor (Molina-Cruz et al., 2020) represent an anchor and an immunity modulator, respectively. The fibrinogen-related domain of FREP1 is able to bind to the acetylglucosamine group, a modification of proteins (Lu et al., 2002). *Plasmodium α*-tubulin-1 has O-linked β-N-acetylglucosaminylation (Kupferschmid et al., 2017) that regulates the assembly and disassembly of the microtubes (Tian, 2019). Our data showed that *Plasmodium* α-tubulin-1 interacts with *Anopheles* FREP1, most likely through the acetylglucosamine group on α-tubulin-1 (Lu et al., 2002). We propose that *Plasmodium* ookinete-expressed α-tubulin-1 at the apical end binds to *Anopheles*-expressed FREP1 protein at the midgut PM, orientating the ookinete invasive apparatus toward the midgut PM (**Figure 10**). The ookinete then secretes digestive enzymes from invasive apparatus (Patra and Vinetz, 2012) to disrupt the integrity of the PM. Both genetic and biochemical (Zhang et al., 2015; Niu et al., 2017) studies show that the FREP1 plays a key, conserved role in the development of malaria parasites in the midgut of multiple *Plasmodium* and *Anopheles* species (Li et al., 2013; Zhang et al., 2015; Niu et al., 2017; Dong et al., 2018). *Plasmodium* α-tubulin-1 is also highly conserved and expressed at all developmental stages. As our data demonstrates, some *Plasmodium* α-tubulin-1 regions are different from human α-tubulin, and therefore are potential targets for blocking parasite development in mosquito hosts.

## Methods

### Expression of candidate proteins using baculovirus in insect cells to verify protein-protein interaction

The full-length coding sequence (CDS) of *P. falciparum HSP70* was amplified with primers of 5’-CGGGATCCATGGCTAACGCAAAAGCAAAGC-3’ and 5’-CGCAAGCTTTTAATCAACTTCTTCAACAGTTGGTC-3’. The full-length CDS of *P. falciparum* 3D7 α-tubulin-1 was amplified with primers of 5’-CGGGATCCATGAGAGAAGTAATAAGTATAC-3’ and 5’-GCTCTAGAATAGTCTGCCTCATATCCTTC-3’. The sequences underlined are restriction enzyme recognition sites. Depending on primer sequences, amplicons were digested with restriction enzymes *BamH*I and *Xba*I or *BamH*I and *HindIII* (New England BioLabs, MA). The column-isolated DNA fragments were ligated into a modified pFastBac1 plasmid having a 6xHis tag at the C-terminus, and positive recombinant plasmids were transformed into DH10Bac competent cells. Recombinant bacmids were extracted from white colonies. The sf9 cells were used to proliferate virus. The high-titer virus was used to infect Hi5 cells to express proteins. Following the manufacturer’s manual (Invitrogen, 2015), 0.5 mL 2x10^5^/mL Sf9 cells in a complete medium (Grace’s medium (ThermoFisher) containing 10% FBS) were deposited into one well of a 24-well plate. After incubation for 30 m, the supernatant was removed. Concurrently, one μL bacmid DNA (1 μg/μL) combined with 25 μL Grace’s medium was mixed gently, and 2 μL Cellfectin II (ThermoFisher) diluted with 25 μL Grace’s medium also was mixed gently. The DNA mixture and Cellfectin II mixture were combined and incubated for 30 m at RT and then added to the above-prepared Sf9 cells. After incubation for 5 h at 27°C, the transfection mixture was removed, and 0.5 ml complete growth medium (Grace’s medium with 10% FBS and 10 units of penicillin/ml and 10 µg/mL streptomycin) was added. After incubation at 27°C for three days, the medium containing recombinant baculovirus particles was collected and used to infect Hi5 to express recombinant proteins. After three passages (10 μl old cell culture to 200 μl new cell culture), expressed proteins were confirmed with ELISA by detecting the His-tag at the C-terminus of recombinant proteins.

### Determination of the interaction between α-tubulin-1 and FREP1 interaction by ELISA and pull-down assays

The Hi5 cell-expressed recombinant α-tubulin-1 proteins were used to determine their specific interaction with FREP1 by ELISA. The expressed proteins in the cell lysate were normalized to 1 nM, and then a 96-well plate was coated overnight with 100 μl cell lysate to detect interactions with FREP1. After blocking the plate with 100 μl 0.2% BSA in 1×PBS, 100 μl of Hi5 cell-expressed recombinant FREP1 (Zhang et al., 2015) in PBS (1 nM) was introduced onto the wells and incubated for 1.5 h at RT. Purified anti-FREP1 Ab (3 μg/mL in PBS) followed by 100 μl 2^nd^ Ab (1:10,000 dilution in PBS) was used to detect any retained FREP1. The plates were developed with 100 µL of *p*-NPP solution (Sigma-Aldrich, St. Luis, MO) until the colors appeared. The absorbance at 405 nm was measured using a plate reader. Between each incubation, wells were washed with 100 µL PBST three times with 3 m incubation each time. For the control group, 1 nM of CAT protein (chloramphenicol acetyltransferase, containing a 6×His tag at the C-terminus) expressed in the same baculovirus-expression system (Thermo Fisher) was used to coat plate wells. The *P. falciparum*-infected blood cell lysate (1 mg/ml proteins) was used to coat wells as the positive control. Each sample was analyzed in triplicate, and the Mann-Whitney test was used to evaluate the difference between the negative control and experimental group.

For the pull-down, we prepared blood-fed mosquito midguts as described previously (Niu et al., 2021). About 400 μl of Hi5 cells lysis (1 mg/mL protein) containing the expressed α-tubulin-1 were mixed with 100 μL of midgut lysis from 100 blood-fed mosquito midguts and then incubated with 100 μL Ni-NTA bead for 24 hours on a rotary shaker (15 rotations per m). After centrifugation (300 g for 5 min), the pellet was washed with PBS three times, and supernatants were discarded. Subsequently, 100 μL SDS-PAGE loading buffer was added, boiled, and spun quickly to settle the beads. The supernatant was used for immunoblotting analysis and reacted with anti-FREP1 antibodies. Hi5 cell lysate expressed with the unrelated protein (CAT) was used as the negative control.

### Culturing *P. falciparum*

*Plasmodium falciparum* parasites (NF54 strain from MR4, Manassas, VA, the catalog is MRA-1000) were maintained in 5 mL RPMI 1640 medium (Life Technologies) supplemented with 10% heat-inactivated (56°C for 45 min) human AB+ serum (Interstate Blood Bank, Memphis, TN), 12.5 μg/ml hypoxanthine, and 4% hematocrit using a 6-well cell culture plate (Corning, NY) in a candle jar at 37°C (Zhang et al., 2015). The parasitemia and gametocytemia were analyzed daily by Giemsa staining of thin blood smears. When the parasitemia reached 5%, 0.5 ml of culture was transferred into 5 ml fresh complete RPMI-1640 supplemented with 4% hematocrit. The remaining culture was maintained for another ten days to two weeks before being used to infect mosquitoes. *P. falciparum* cultures harboring 2-5% stage V gametocytes were diluted 10-fold in RPMI 1640 supplemented with 20% heat-inactivated human AB+ serum and 50 μg/mL hypoxanthine and incubated at room temperature for 18-24 h to develop ookinetes.

### Determine the localization of-tubulin-1 on live *P. falciparum* ookinete surface by IFA

IFAs were conducted independently with rabbit polyclonal Ab against human α-tubulin (ProteinTech, IL, USA) and against *P. falciparum* α-tubulin-1. Ab was labeled with CF™ 568 dye (Mix-n-Stain™ CF^®^ Dye Antibody Labeling Kits - CF^®^568, Biotium Inc, CA). The cultured *P. falciparum* ookinete cell mixtures (10^5^ cells) were suspended at 200 µL in a complete RPMI-1640 medium and were deposited carefully on the top of 0.6 mL 65% Percoll (100% Percoll diluted with incomplete RPMI-1640) in a 1.5 mL plastic tube. After centrifugation at 2,000 rpm for 5 m, the cells at the interface of medium and 65% Percoll were collected and diluted 10-fold with incomplete RPMI-1640 medium and then collected by centrifugation at 2,000 rpm for 5 m. The cells were washed twice with 0.5 mL PBS through centrifugation (2,000 rpm for 5 m). Because the fluorescence is sensitive to light, the following steps were done in a dark room. The cells were suspended in 50 µL PBS and incubated with fluorescence-conjugated anti-α-tubulin Ab (200-fold dilution, final Ab concentration was ~1 µg/mL) for 45 m. The cells were collected and washed with 0.5 mL PBS twice by centrifugation (2,000 rpm for 5 m). Finally, the cells (~10^4^ cells) were suspended in 20 µL PBS, and 4 µL were deposited onto a slide coverslip. Immediately after drying, the cells on a coverslip were mounted on a slide with 5 μL of Vectashield mounting media (Vector Laboratories, Burlingame, CA). After incubation at 4°C for 2 h, the cells were examined using a Nikon Eclipse Ti-S fluorescence microscope. Cells treated similarly except using unrelated antibodies (anti-V5) were used as a negative control. Cells on slides were fixed with methanol and incubated with fluorescence-conjugated anti-α-tubulin Ab (200-fold dilution, final Ab concentration was ~1 µg/mL) as a positive control.

### Antibody transmission-blocking assays of *P. falciparum* infection in *An. gambiae*

SMFA was performed as described (Niu et al., 2015; Zhang et al., 2015). The commercial rabbit polyclonal Ab against full-length human α-tubulin or β-tubulin (ProteinTech, IL, USA) were used in the malaria transmission-blocking assay. Approximately 6μL rabbit polyclonal Ab against human *α*-tubulin (0.3 mg/mL) was added into 200 μL *P. falciparum*-infectious blood (final Ab concentration was about 0.01 mg/mL) and fed to *An. gambiae*. The same amount of non-related purified rabbit pAb (anti-V5 tag) was used as a control. The number of oocysts in mosquito midguts was counted seven days late and analyzed statistically by the Mann-Whitney test that was implemented in GraphPad Prism software (Version 6h, San Diego, CA).

We commercially generated specific pAb against *P. falciparum* α-tubulin-1 (Boster Bio, Pleasanton, CA). The full-length *P. falciparum α-tubulin-1* and *Hsp70* genes were cloned from *P. falciparum* 3D7 and expressed in *E. coli*. The *P. falciparum* 3D7 *Hsp70* gene was chosen as a negative control simply because it is also an abundant cytoplasmic protein. The pAb against *P. falciparum* α-tubulin-1 was generated in rabbits. The anti-sera were normalized with a pre-immune serum to the titer of 2x10^5^, mixed with the same volume of cultured *P. falciparum*, and fed 3-5-day-old female *An. gambiae*. The number of oocysts in the mosquito midguts was analyzed as described previously.

The rabbit pAb against *P. falciparum* 3D7 α-tubulin-1 was purified through protein A/G column and used in SMFA to rule out the possible effect of other components in sera. About 6μL rabbit pAb with different concentrations was mixed with 200 μL *P. falciparum*-infectious blood (final Ab concentrations were 0, 17, 50, 150, 450 μg/mL) and fed to *An. gambiae*. Seven days late, the number of oocysts in mosquito midguts was counted for statistical analysis by the Mann-Whitney test.

The half maximal inhibitory concentration (IC_50_), e.g., the inhibition concentration that displayed 50% of infection intensity (the number of oocysts), was calculated using an online tool (APA, 2023).

We also generated mouse monoclonal antibodies (mAb) using *E. coli*-expressed recombinant full-length *P. falciparum* α-tubulin-1 through Boster Bio (Pleasanton, CA). The mAb were purified using protein A/G.

### Ookinete conversion assays in the presence of Ab

First, the numbers of gametocytes in cultures were counted in a blood smear under light microscopy. Approximately 1 mL of 15-day *P. falciparum*-cultured blood that harbors stage V gametocytes was collected by centrifugation (800x g for 4 m) and re-suspended into 10-fold ookinete cultures (RPMI-1640 supplemented with 20% heat-inactivated human AB+ serum and 50 μg/mL hypoxanthine). Approximately 3 µL and 5 µL of polyclonal anti-human α-tubulin Ab (0.3 mg/mL, Abcam) was then added into 150 µL *P. falciparum*-infectious blood (50- and 30-fold dilutions, final Ab concentrations were ~6 µg/mL and 10 µg/mL respectively) to a well in a 96 well plate. The parasites were then incubated at 21-23°C for about 16 hours. The same amount of non-related purified rabbit Ab (anti-V5) was used as a control. The number of ookinetes was counted, and the ookinete conversion rate (CR), the percentage of ookinetes among the total *P. falciparum* gametocytes, was calculated.

### Mapping monoclonal epitopes by peptide arrays

We mapped epitopes commercially (Pepperprint, Germany). A custom peptide microarray was pre-stained with the secondary antibody to investigate background interactions with the antigen-derived peptides that could interfere with the main assays. Subsequent incubation of further custom peptide microarrays with mouse IgG antibodies at concentrations of 1 µg/ml, 10 µg/ml, and 100 µg/ml was followed by staining with the secondary and control antibody. Read-out was performed with an Innopsys InnoScan 710-IR Microarray Scanner. The additional HA control peptides framing the peptide microarrays were subsequently stained with the control antibody as internal quality control to confirm assay performance and peptide microarray integrity. Microarray image analysis was done with PepSlide^®^ Analyzer. The intensity plots were correlated with peptides on the microarray to identify the epitopes of the antibody samples. For a better data overview, the baselines of the intensity plots were shifted up.

## Data availability statement

The original contributions presented in the study are included in the article/supplementary material. Further inquiries can be directed to the corresponding author.

## Author contributions

GZ started the project as his dissertation and made the initial discovery. GN conducted antibody-blocking assays and IFA. JR conducted Western blotting and confocal IFA. SS analyzed the specificity of antibodies. XW analyzed sequences and infection assays. NB participated in the experimental design and data interpretation. AJ interpreted data and edited the manuscripts. GZ and JL wrote the manuscript. JL conceived the study. All authors contributed to the article and approved the submitted version. Thank Laura Perez for help in conducting infection assays.
